# Chitobiose exhibited a lipid-lowering effect in ob/ob^−/−^ mice via butyric acid enrolled liver–gut crosstalk

**DOI:** 10.1186/s40643-023-00696-7

**Published:** 2023-11-09

**Authors:** Xinye Zhuang, Mengyao Zhao, Xiaoguo Ji, Sihan Yang, Hao Yin, Liming Zhao

**Affiliations:** 1 State Key Laboratory of Bioreactor Engineering, School of Biotechnology, East China University of Science and Technology, Shanghai, 200237 China; 2 Organ Transplant Center, Shanghai Changzheng Hospital, Shanghai, 200003 China; 3 Shanghai Collaborative Innovation Center for Biomanufacturing Technology (SCICBT), Shanghai, 200237 China

**Keywords:** Chitobiose, Butyric acid, Non-alcoholic fatty liver disease, Lipid β-oxidation, Liver–gut axis signaling

## Abstract

Chitobiose (COS_2_) efficiently lowers lipids in vivo and facilitates butyric acid enrichment during human fecal fermentation. However, whether COS_2_ can interact with butyric acid to generate a hypolipidemic effect remains unclear. This study examined the hypolipidemic mechanism of COS_2_ involving butyric acid, which could alleviate non-alcoholic fatty liver disease (NAFLD). The results revealed that COS_2_ administration modulated the β-oxidation pathway in the liver and restructured the short chain fatty acids in the fecal of ob/ob^−/−^ mice. Moreover, the hypolipidemic effect of COS_2_ and its specific accumulated metabolite butyric acid was verified in sodium oleate-induced HepG2 cells. Butyric acid was more effective to reverse lipid accumulation and up-regulate β-oxidation pathway at lower concentrations. Furthermore, structural analysis suggested that butyric acid formed hydrogen bonds with key residues in hydrophilic ligand binding domains (LBDs) of PPARα and activated the transcriptional activity of the receptor. Therefore, the potential mechanism behind the lipid-lowering effect of COS_2_ in vivo involved restoring hepatic lipid disorders via butyric acid accumulation and liver–gut axis signaling.

**Graphical Abstract:**

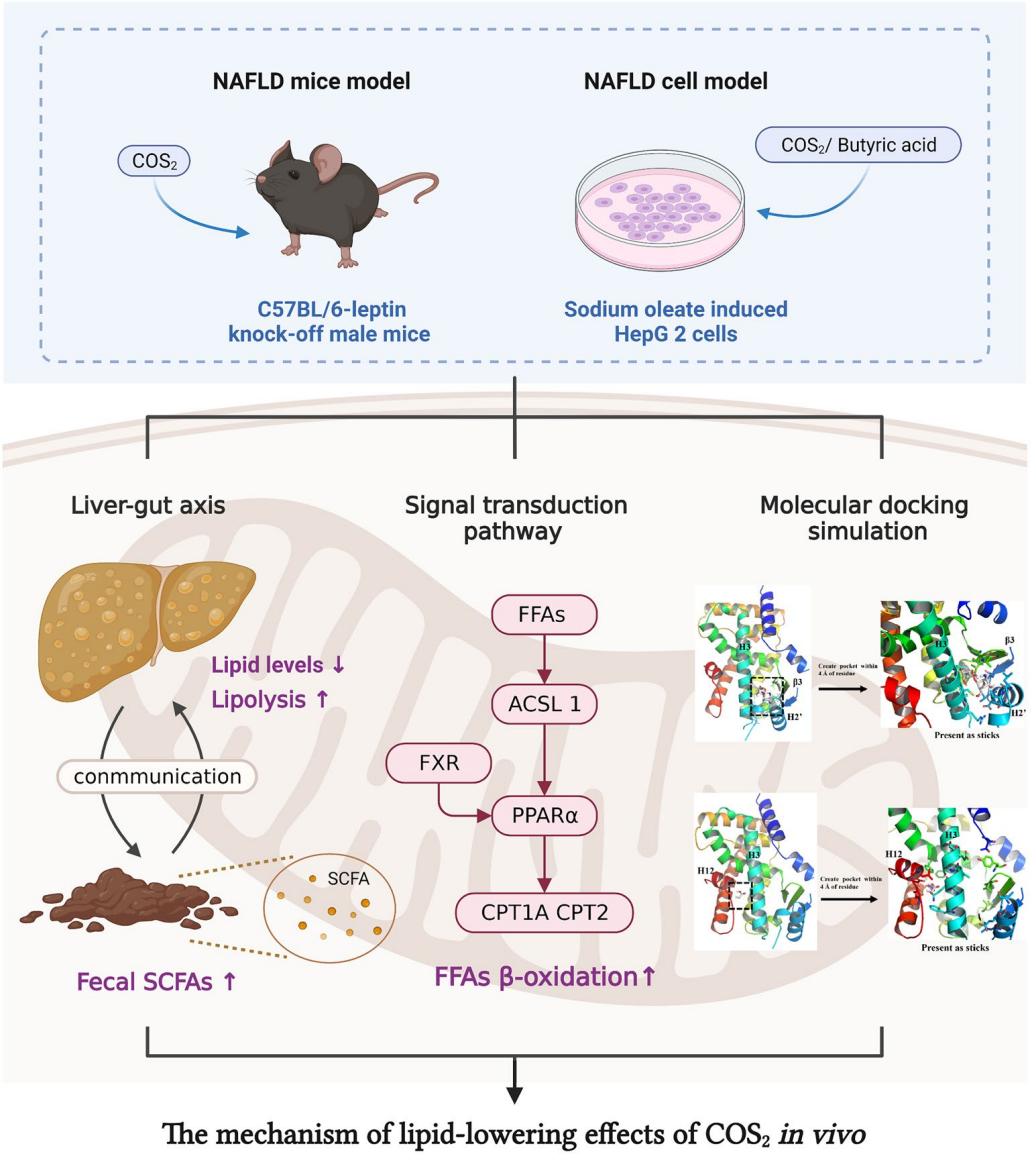

**Supplementary Information:**

The online version contains supplementary material available at 10.1186/s40643-023-00696-7.

## Introduction

Non-alcoholic fatty liver disease (NAFLD) is defined as lipid accumulation (more than 5%) in hepatocytes without alcohol consumption, viral infection, or other known pathogenic factors. NAFLD includes simple lipid steatosis, non-alcoholic steatohepatitis (NASH), and advanced fibrosis (Vernon et al. 2011) and is the leading cause of liver disease with a prevalence of 25.24% worldwide (Younossi et al. 2016) and 29.2% in China (Zhou et al. 2019). However, the pharmaceutical therapies available for NAFLD clinical treatment are limited due to complex and diverse etiologies and extended disease development. Therefore, treating and preventing NAFLD and chronic liver disease depend on the availability of safe, effective, and diverse therapeutic agents, the development of which is crucial.

Chitosan oligosaccharides (COS) are chitosan degradation products that can regulate body weight and lipid metabolism by modifying the dysfunctional gut microenvironment (He et al. 2020; Wang et al. 2020). Previous studies have shown that COS reduces the intracellular triglyceride (TG) levels in oleic acid-induced HepG2 cells (Cao et al. 2016), displays anti-obesity activity, and improves serum and liver lipid profile abnormalities in high-fat diet-induced C57BL/6N mice (Choi et al. 2012; Li et al. 2022a, b), highlighting the potential of COS in preventing NAFLD. Studies have increasingly focused on the molecular COS mechanism involved in the hypolipidemic effect, including lipid uptake regulation, de novo synthesis, and free fatty acid (FFAs) β-oxidation (Liu et al. 2021; Tao et al. 2019; Zheng et al. 2018). The dimer COS is one of the most promising COS monomers (*DP* = 2 ~ 6) for alleviating lipid accumulation by decreasing the hepatic lipid uptake and de novo synthesis in oleic acid-induced HepG2 cells (Li et al. 2018). Shen et al. demonstrated that COS_2_ and COS_3_ reversed dyslipidemia by reducing the hepatic FFAs’ uptake and de novo synthesis of TG by downregulating the mRNA and protein levels of CD36 and DGAT2 in ob/ob^−/−^ mice (Shen et al. 2021). The regulation of FFAs β-oxidation is also essential for limiting lipid accumulation. The main β-oxidation pathways are as follows: firstly, FFAs were activated and transferred to fatty acyl-CoA by ACSL1 in the outer mitochondrial membrane. Then, carnitine palmitoyl transferase 1A (CPT1A) and carnitine palmitoyl transferase 2 (CPT2), stimulated by PPARα, promote fatty acyl-CoA to cross the mitochondrial membrane and oxidized in the mitochondrial matrix finally under the effects of oxidation enzymes including PPARα (Huh et al. 2020). However, the potential intervention mechanism of COS_2_ during β-oxidation remains unclear.

Focusing on lipid metabolism regulation is insufficient to elucidate the mechanism behind NAFLD alleviation. Studies have suggested that the "multiple parallel hit" is more suitable for NAFLD pathogenesis (Buzzetti et al. 2016; Tilg et al. 2021). The cause of NAFLD includes hepatic lipid metabolism disruption and is strongly associated with the alteration of gut flora and their metabolic products (Li et al. 2022a, b). Therefore, the interaction and communication of the liver–gut axis cannot be disregarded during NAFLD intervention. COS reportedly prevents metabolic syndrome by promoting the growth of beneficial intestinal bacteria and decreasing the abundance of inflammogenic taxa in HFD mice (Qian 2019). Additionally, gut metabolite alteration is crucial in the NAFLD intervention mechanism. Research has shown that oral supplementation with butyric acid protects mice and rats from hepatic inflammation and lipid steatosis (Hattori et al. 2022; Jin et al. 2015; Sun et al. 2018; Zhao et al. 2021). Therefore, this study hypothesizes that the effects of COS_2_ in NAFLD were under the promotion of butyric acid in vivo. Butyric acid then circulates into the liver via the enterohepatic axis, playing a role in restoring lipid metabolism.

The objective of this study is to elucidate whether COS_2_ is involved in the liver–gut axis to alleviate NAFLD and the role the primary secondary metabolite, butyric acid, plays in COS_2_ enrolled intervention. NAFLD mice and cell models were established to evaluate the anti-hyperlipidemic effect of COS_2_ and butyric acid. Additionally, the gene and protein levels of the targets related to the β-oxidation pathway were explored to reveal the intervention mechanism. Results will indicate whether the anti-NAFLD effect of COS_2_ is positively related to butyric acid, which mainly contributes to β-oxidation activation.

## Methods

### Materials

Briefly, highly efficient and specific chitosanase was developed and used to obtain a series of COS with different degrees of polymerization (Luo et al. 2020). These products were then separated and desalted via ultrafiltration and nanofiltration to produce COS_2_ (purity > 95%, degree of deacetylation > 95%). The corresponding liquid phase diagram is shown in Additional file 1: Figures S1.

The sodium butyrate (NaB) was purchased from Sigma-Aldrich Chemical (St. Louis, MO, USA), while the tyrisin was obtained from Haoyang Biological Manufacture Co., Ltd. (Tianjin, China). The TG, total cholesterol (TC), low-density lipoprotein cholesterol (LDL-C), and high-density lipoprotein cholesterol (HDL-C) assay kits were provided by the Nanjing Jiancheng Bioengineering Institute (Nanjing, China). The total RNA isolation kit and HiScript^®^ III RT SuperMix for the quantitative polymerase chain reaction (qPCR) (+ gDNA wiper) were purchased from Vazyme Biotech Co., Ltd (Nanjing, China). Mei5 Biotechnology, Co., Ltd (Beijing, China) provided the 2X M5 HiPer SYBR Premix EsTaq (with Tli RNaseH), while the RIPA lysis buffer was acquired from BioSharp (Anhui, China). The BCA Protein Assay Kit was purchased from CoWin Biosciences (CWBIO). The CPT1A, CPT2, and GAPDH polyclonal antibodies were acquired from the Proteintech Group, Inc (USA), while the secondary goat-anti-rabbit IgG-HRP antibodies were provided by Abmart, Inc (Shanghai, China). All reagents were analytically pure and obtained from commercial suppliers.

### Animal models

Specific-pathogen-free (SPF) C57BL/6-leptin knock-off male mice (4 weeks old), in an initial weight range of 21 ~ 25 g, were purchased from Jiangsu Jicui Yaokang Biotechnology Co., Ltd. (Animal Certificate No. 201908218). The animal treatments involved in this study and their experiments were conducted in accordance with the Animal Research: Reporting of In Vivo Experiments (ARRIVE) guidelines and the National Research Council's Guide for the Care and Use of Laboratory Animals. These experiments had been reviewed by the Experimental Animal Ethics Committee of Jiangnan University (JN. No20190930b0560215). All animals were housed in standard laboratory conditions (22 °C $\pm$ 3 °C, 12 h light–dark cycle) with free access to clean drinking water while receiving a standard diet (XieTongShengWu Ltd, Nanjing, Jiangsu, China, no. 101009) for ten weeks. COS_2_ were dissolved with 0.9% NaCl, while all the solutes were dissolved to the same volume. After an adaptation phase of one week, the animals were randomly divided into three groups (n = 8): The model group was gavaged with 0.9% NaCl as a solvent control. The other two groups received different COS_2_ dosages of 500 mg kg^−1^ day^−1^ (COS_2_-H) and 250 mg kg^−1^ day^−1^ (COS_2_-L) via intragastric administration. During the intervention, the body weights were measured. At the end of the experiment, all the animals were killed after an 18-h fasting period. Blood samples were collected and centrifuged at 106 *g*/min for 15 min at 4 °C to separate the serum, after which the liver tissue was collected and weighed. The biological samples were stored at −80 °C for further examination.

### Analysis of fecal SCFAs

To determine the contents of SCFAs in mouse fecal samples, 50 mg of fecal sample was weighed accurately and mixed vigorously with 400 μL of sterile water. Then, 50 μL of 50% sulfuric acid was added for sufficient acidification, followed by the addition of 200 μL of ethyl acetate. The mixture was vortexed vigorously and allowed to stand for 2 min. Afterward, it was centrifuged at 8000 *g* and low temperature for 15 min, and the upper organic phase was collected for SCFAs quantitative analysis. SCFAs were determined by gas chromatography using an Agilent 7890 A gas chromatograph equipped with a flame ionization detector (FID) (California, United States) and an HP-5MS polar column (0.25 μm × 0.25 mm × 30 m). The temperature program was set as follows (Ji et al. 2022): the initial column temperature was 140 °C for 10 min, then ramped at a rate of 5 °C/min to 165 °C and held for 2 min, and finally ramped at a rate of 25 °C/min to 270 °C and held for 2 min. The detector temperature was set at 280 °C, and the injection port temperature was set at 250 °C. The qualitative and quantitative analysis of SCFAs, including acetic acid, propionic acid, and butyric acid, were performed using the standard curve method.

### Cell culture

HepG2 cells were cultured in Minimum Essential Medium (Gibco, Grand Island, NY) with 10% fetal bovine serum (Gibco, Grand Island, NY), 1% penicillin–streptomycin solution (Haoyang Biological Manufacture Co., Ltd., Tianjin, China), non-essential amino acids, and sodium pyruvate solution (Sigma-Aldrich Chemical, St. Louis, MO). The cells were incubated at 37 °C in a 5% CO_2_ atmosphere.

Sodium oleate was used to induce NAFLD cell model with the concentration of 0.01 mM. The application of COS_2_ (0.02 mΜ, 0.2 mΜ, 0.5 mM, 1 mM) or NaB (0.01 mM, 0.1 mM, 0.3 mM, 0.5 mM) was undertaken into NAFLD cell model. After 24 h of incubation, cells were harvested and samples were prepared.

### Biochemical indicators and histological analysis

Enzyme-linked immunosorbent assay (ELISA) kits were used to determine the lipid indicators of serum and liver of mice. The blood samples were centrifuged at 106 *g* for 15 min and the supernatant was collected and stored at −80 °C. The liver tissues were accurately weighed, mixed with a ninefold weight of ethanol, and homogenized at 4 °C for 120 s (60 Hz) to obtain the 10% homogenate. A commercial kit was used to analyze the TG, TC, LDL-C, and HDL-C levels in the serum and liver tissue samples according to the standard protocols. A histological section of the liver and HepG2 cells were immersed and dyed with Oil Red O solution to detect the lipid accumulation. Photos were taken at 400 × magnification using a Nikon Eclipse TI fluorescent microscope (Nikon, Tokyo, Japan).

### Real-time PCR

The total RNA was extracted from the liver and HepG2 cells using a total RNA isolation kit according to the instructions of the manufacturer. The total RNA was reversely transcribed into cDNA using HiScript^®^ III RT SuperMix. The qPCR primers were synthesized by Sangon Biotech (Shanghai, China), and the sequences are listed in Additional file 1: Table S1. The qPCR mixture was prepared using 2 × M5 HiPer SYBR Premix EsTaq (with Tli RNaseH), while the reaction was performed via CFX96 Touch Real-Time PCR (Bio-Rad, CA, USA). All procedures were conducted according to the instructions of the manufacturers. The Cycle threshold (C_t_) values of each target gene were obtained and calculated after normalizing the housekeeping gene at 2^−ΔΔct^.

### Western blot

The liver tissues and cells were lysed and homogenized at 4 °C using RIPA lysis buffer, phosphatase inhibitors, and protease inhibitors. The homogenate was centrifuged at 15,000 *g* for 15 min at 4 °C. The protein concentration was determined using a BCA protein assay kit. The protein samples were separated via 10% SDS-PAGE and then transferred to a polyvinylidene difluoride (PVDF) membrane (Merck Millipore). The membrane was blotted in Tris-buffered saline with tween 20 (TBST) containing 5% skim milk for 1 h at room temperature. Then, the polyclonal antibodies were diluted to 1:1000 with primary antibody dilution buffer, and the membranes were incubated overnight at 4 °C, followed by incubation with horseradish peroxidase-conjugated secondary antibodies (1:3000) for 1 h. The target bands were scanned using a Tannon automatic chemiluminescence image analysis system and examined via Image J software.

### Molecular docking simulation

The PPARα protein (PDB code: 2ZNN) was downloaded on Protein Data Bank (RCSB PDB: Homepage). The original ligand was removed by PyMOL (Version 2.1.1_0) to prepare PPARα crystal structure. The molecule structure of COS_2_ and butyric acid were mapped using ChemBio3D Ultra 14.0. The ligand docking center and the size of the grid box were determined on the native ligand and obtained by AutoDockTools 1.5.6. The docking studies were performed by AutoDock Vina 1.1.2 (Trott et al. 2010). The results were analyzed by PyMOL.

### Statistical analysis

The experimental data were generated with GraphPad Prism 8.0 (GraphPad Software, San Diego, USA) and expressed as mean ± SD. The Oil Red O staining images were analyzed using Image J statistical software via one-way ANOVA with multiple comparisons to show the differences between groups. *P*  < 0.05 were considered significant.

## Results

### ***COS***_***2***_*** decreased lipid deposition in the serum and liver of ob/ob***^***−/−***^*** mice***

**Fig. 1:**
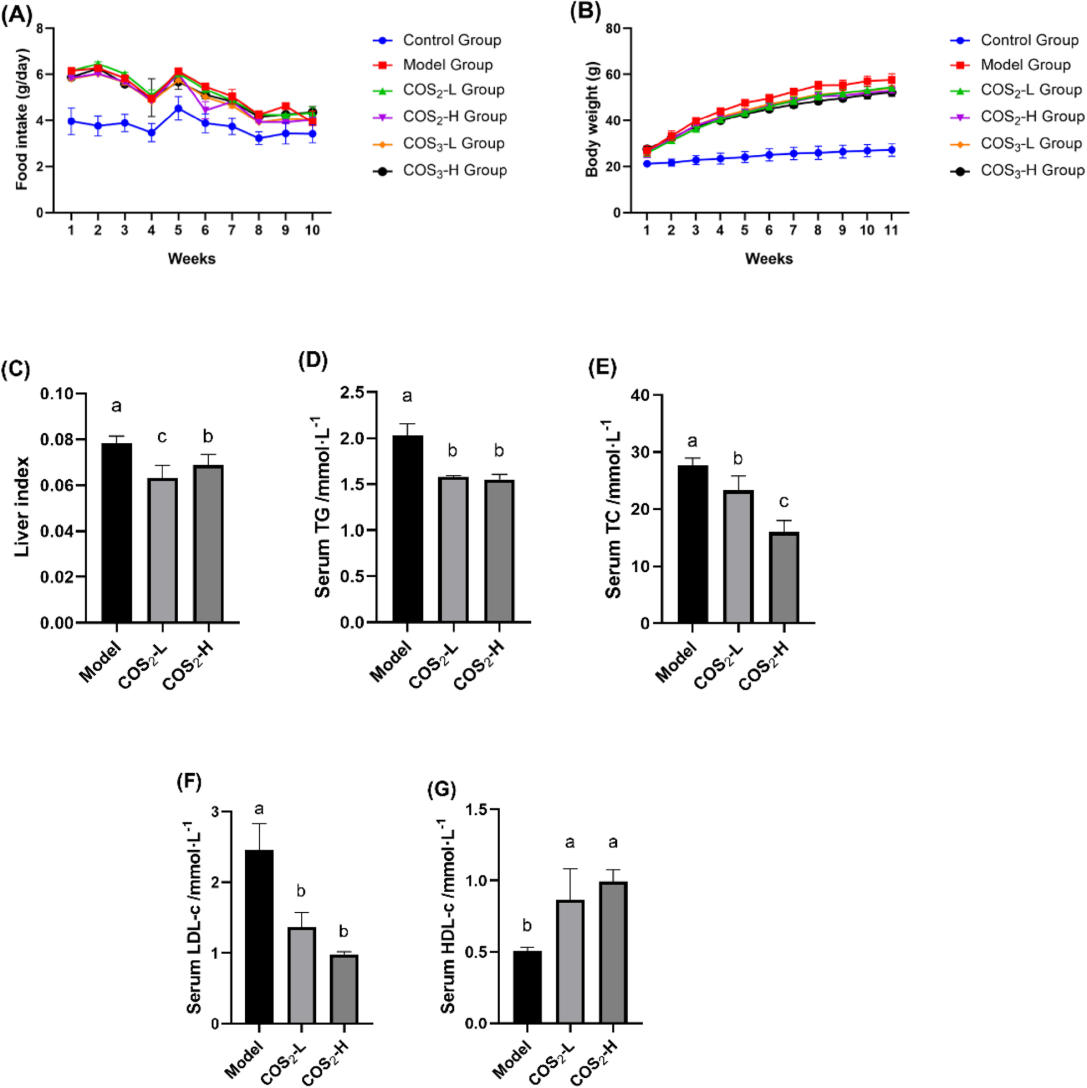
The improvement of lipid accumulation induced by COS_2_ in ob/ob^−/−^ mice. **A** Body weight. **B** Food intake. **C** Liver index. **D** Serum TG. **E** Serum TC. **F** Serum LDL-C. **G** Serum HDL-C

**Fig. 2:**
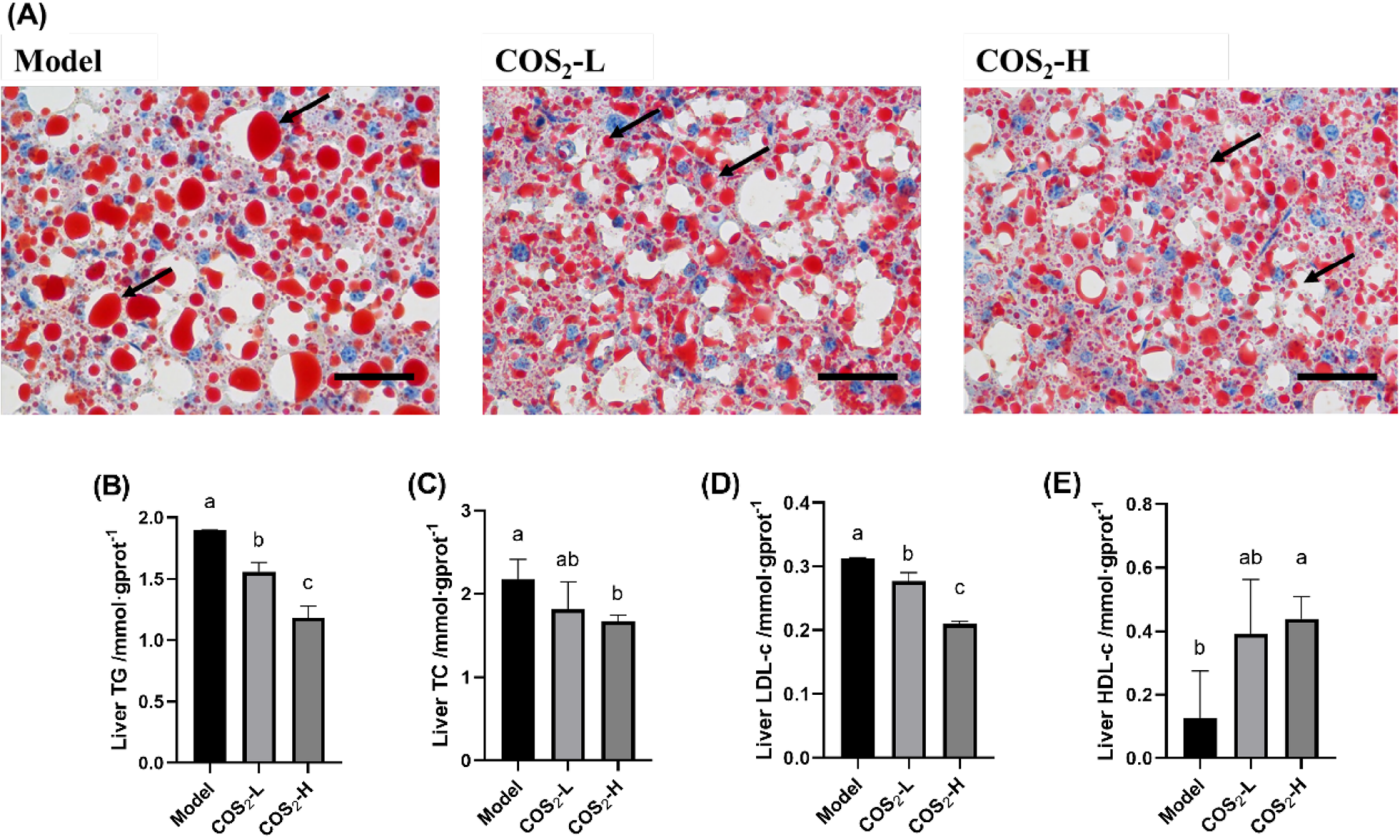
The effect of COS_2_ on lipid accumulation amelioration in the liver of the ob/ob^−/−^ mice. **A** Oil Red O staining (400$\times$magnification, scale bar = 200 µm). The arrows indicate the lipid droplet size in the liver. **B** Hepatic TG. **C** Hepatic TC. **D** Hepatic LDL-C. **E** Hepatic HDL-C

Ten weeks of COS_2_ intervention distinctly reduced the body weight gain of the ob/ob^−/−^ mice (Fig. 1A, p < 0.05) and decreased the liver index compared with the model group (*p* < 0.05) (Fig. 1B). The TG, TC, and LDL-C levels in the serum were improved after COS_2_ intervention (Fig. 1C, D, E), while the HDL-c levels increased (Fig. 1F, p < 0.05). Moreover, the lipid droplets in the liver were smaller in the COS_2_ treatment groups than in the model group (Fig. 2A). The lipid profiles in the liver, including TG, TC, and LDL-C, decreased while the HDL-C level increased after COS_2_ administration (Fig. 2B, C, D, E, p < 0.05). These results indicated that COS_2_ ameliorated lipid deposition in the serum and liver of ob/ob^−/−^ mice.

### ***COS***_***2***_*** regulated lipid metabolism to promote the lipid β-oxidation levels of ob/ob***^***−/−***^***mice***

**Fig. 3:**
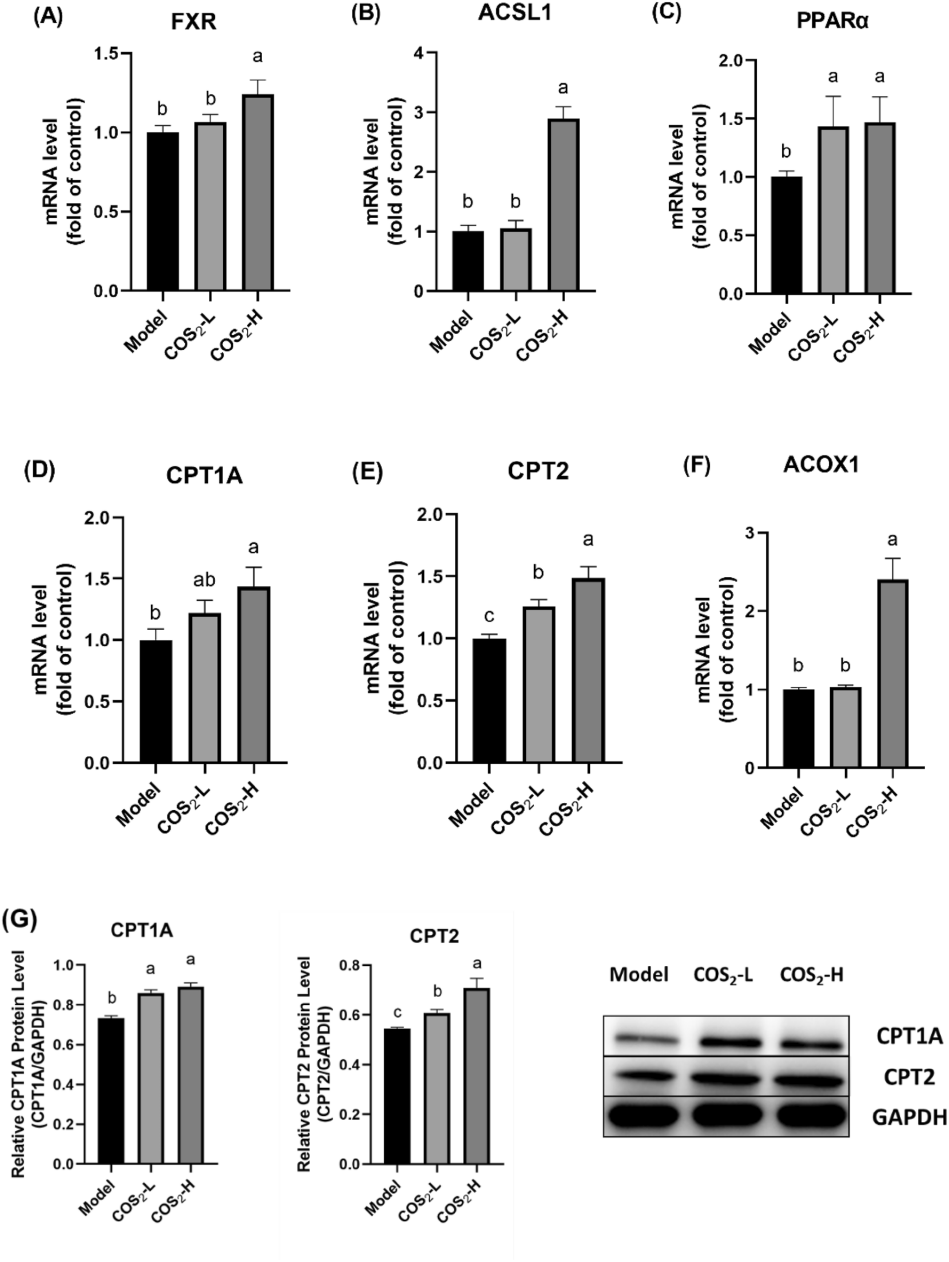
The mRNA levels of **A** FXR, **B** ACSL1, **C** PPARα, **D** CPT1A, **E** CPT2, and **F** ACOX1, and the protein levels of **G** CPT1A and CPT2, induced by COS_2_ in targets related to lipid oxidation in the livers of the ob/ob^−/−^ mice

Farnesoid X receptor (FXR) is crucial for lipid homogenesis and protects the liver from lipid accumulation and hepatic steatosis (Schmitt et al. 2015). This study showed a distinct increase in the hepatic FXR gene expression level in the ob/ob^−/−^ mice at high COS_2_ gavage concentrations (Fig. 3A, p < 0.05), accounting for the inhibition of lipid uptake and synthesis shown by the previous results (Shen et al. 2021), and contributing to FFA β-oxidation upregulation (Xi et al. 2020). Although the β-oxidation pathways helped eliminate excessive FFAs, the role of COS_2_ in FFAs β-oxidation regulation remains unclear. Acyl-CoA synthetase long-chain family member 1 (ACSL1) is an enzyme located in the outer mitochondrial membranes, responsible for transferring fatty acids to acyl-CoA (Coleman et al. 2000; Huh et al. 2020). High-dose COS_2_ treatment significantly increased the ACSL1 mRNA levels in the livers of the ob/ob^−/−^ mice (Fig. 3B, p < 0.05). PPARα, induced by FXR, is a transcription factor that regulates FFAs β-oxidation (Fuchs et al. 2016; Sinal et al. 2001), the gene expression of which was increased by COS_2_ treatment (Fig. 3C, p < 0.05). As the downstream target of PPARα, CPT1A and CPT2 represent essential regulators for FFA translocation into the mitochondria, denoting the rate-limiting steps of mitochondrial β-oxidation (Xi et al. 2020). COS_2_ significantly increased the mRNA levels and CPT1A and CPT2 protein content in a dose-dependent manner (Fig. 3D, E, G, p < 0.05). Furthermore, ACOX1 represents the first enzyme in the FFA β-oxidation pathway. High-dose COS_2_ treatment upregulated the ACOX1 gene level, facilitating FFA oxidation in the liver (Fig. 3F). Therefore, COS_2_ accelerated FFA conversion and translocation in the liver and increased the mitochondrial β-oxidation rate.

### ***COS***_***2***_*** regulated the synthesis of SCFAs, leading to a significant increase in the production of butyrate in ob/ob***^***−/−***^*** mice***

**Fig. 4:**
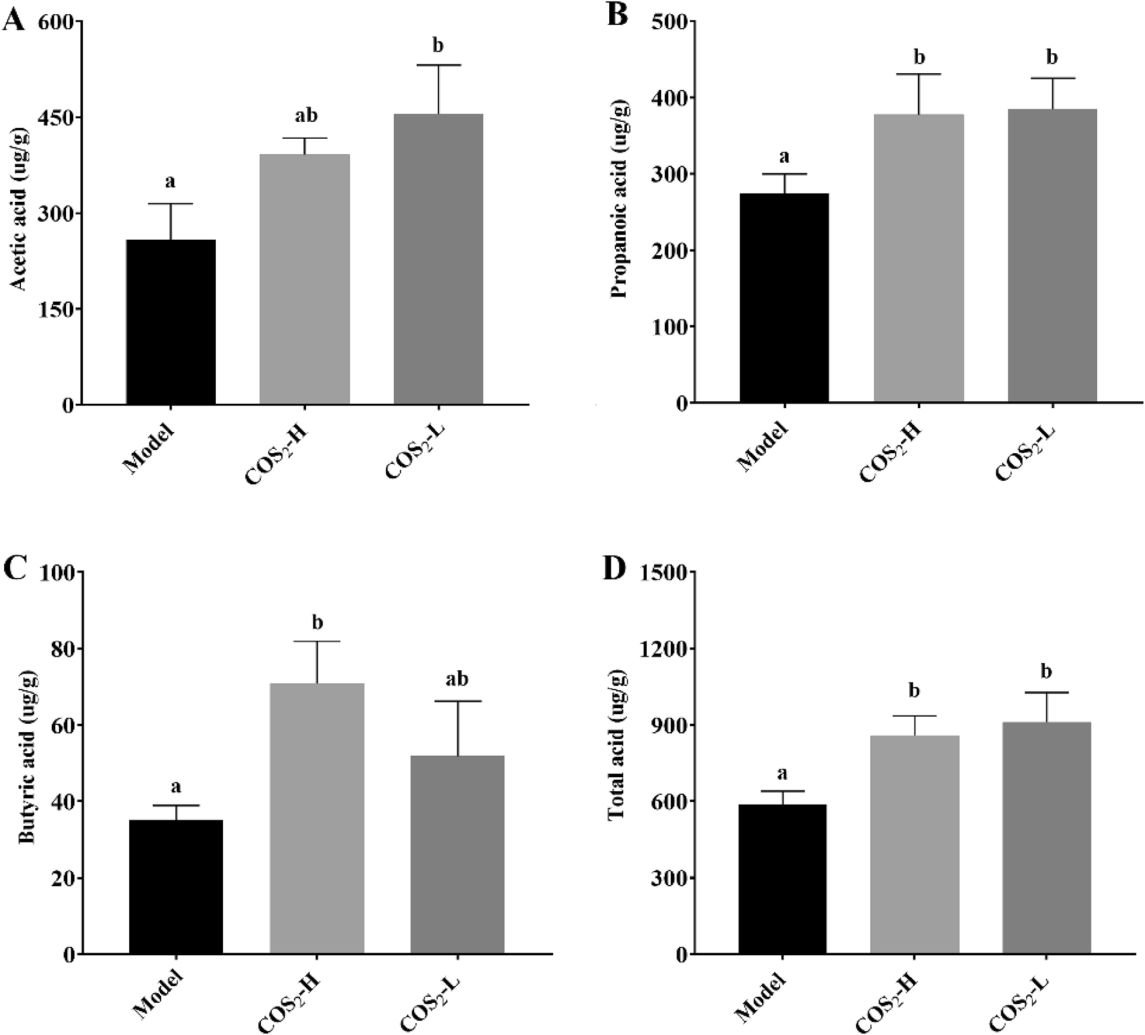
Changes in SCFAs in the colon of ob/ob^−/−^ mice treated with different doses of COS_2_. **A** Acetic acid. **B** Propionic acid. **C** Butyric acid. **D** Total SCFAs

Butyric acid is an SCFAs fermented from dietary fibers by gut microbiota in the colon. Differential analysis was performed on the content of SCFAs in the feces of mice from different treatment groups (Fig. 4). Acetic acid and propionic acid serve as substrates for hepatic gluconeogenesis, and enter the hepatic portal system through intestinal absorption, promoting hepatic gluconeogenesis metabolism and providing energy for liver metabolism. Compared with the model group (Fig. 4A), the acetic acid content in the COS_2_ high and low dose groups increased significantly (*p* < 0.05). The propionic acid content in the COS_2_ high and low dose groups increased significantly (Fig. 4B, p < 0.05). Butyrate is the energy source for intestinal epithelial cells and can have a preventive and therapeutic effect on NAFLD and T2DM. Compared with the model group, the butyric acid content in the COS_2_ high-dose group increased significantly (Fig. 4C), which is consistent with the results of previous reports on the changes in gut microbiota induced by COS_2_ (Ji et al. 2021, 2022). Analysis of the total SCFAs content in the intestine of COS-intervened model mice revealed a significant increase in the COS_2_ treated group.

Recent studies have found that butyric acid can exhibit functions and impact other tissues and organs beyond the gut via the enterohepatic circulation (van der Hee et al. 2021). Our previous research showed that COS_2_ enhanced butyric acid accumulation by promoting the abundance of *Clostridium_sensu_stricto_1*, *Clostridium_sensu_stricto_13,* and *Fusobacterium* (Ji et al. 2021, 2022). Moreover, butyric acid decreased lipogenesis to alleviate PPARγ in the liver and reversed lipid accumulation via the liver–gut axis (den Besten et al. 2015). Therefore, it can be considered a potential hypolipidemic biomarker during COS_2_ metabolism in the colon. The role of COS_2_ during FFAs β-oxidation was correlated with butyric acid to determine its potential lipid-lowering mechanism in conjunction with COS_2_.

### ***The hypolipidemic effect of COS***_***2***_*** and the typical metabolite modulated by COS***_***2***_*** in sodium oleate-induced HepG2 cells***

**Fig. 5:**
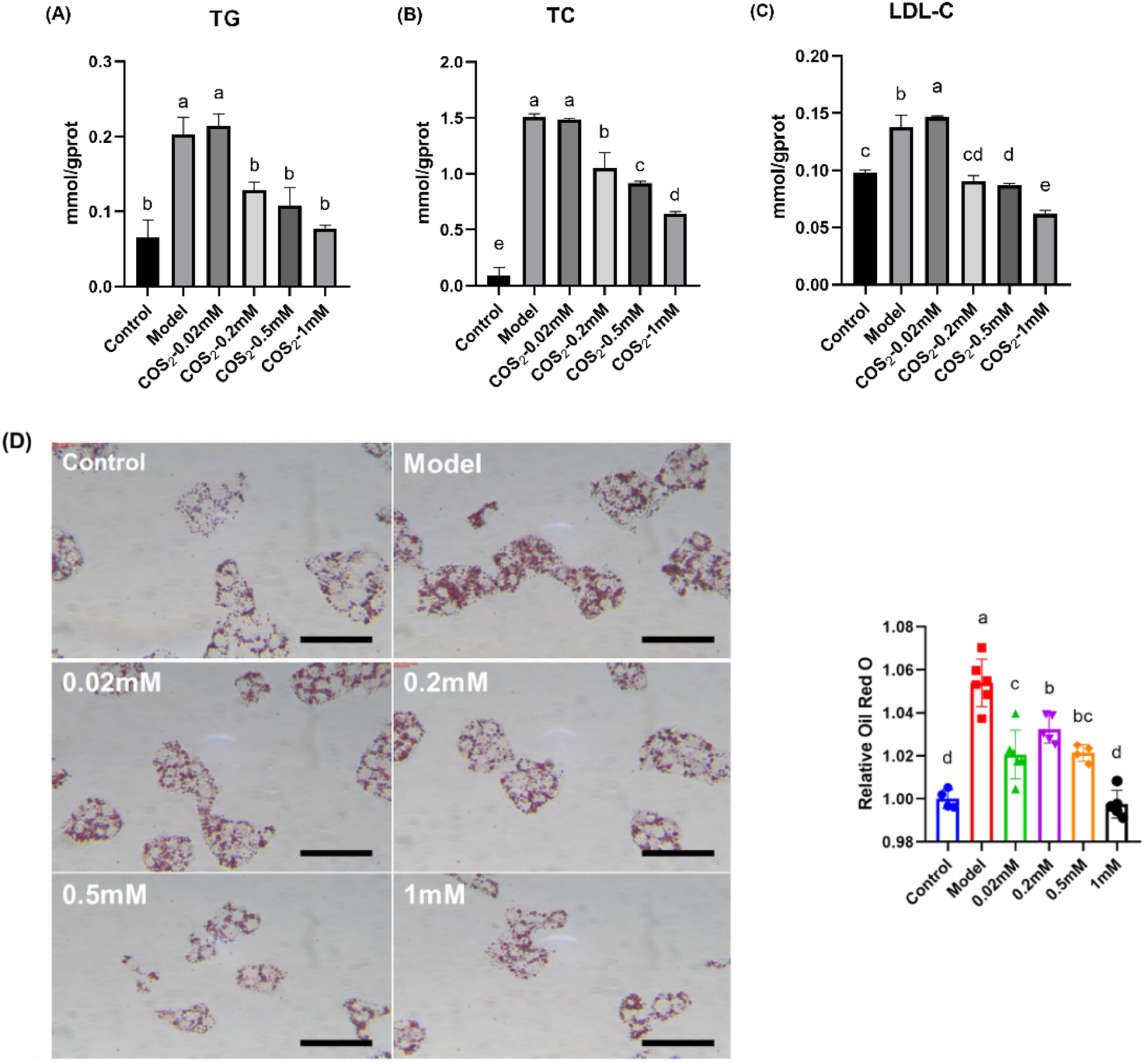
The effect of different COS_2_ concentrations on the lipid levels in oleic acid-induced HepG2 cells. **A** TG levels. **B** TC levels. **C** LDL-C levels. **D** Lipid accumulation after Oil Red O staining (scale bar = 500 nm)

**Fig. 6:**
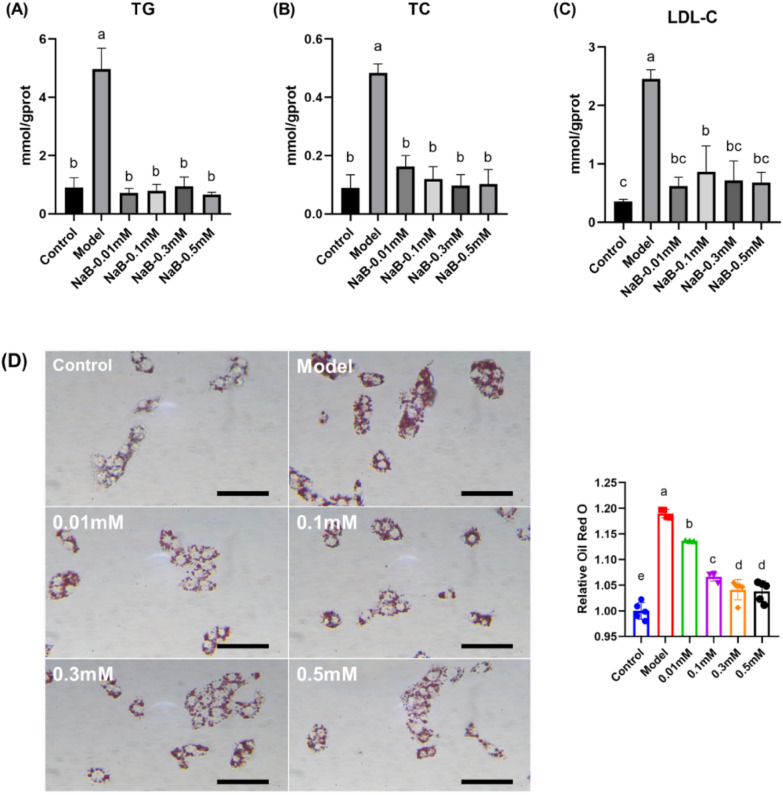
The effect of different NaB concentrations on the lipid levels in sodium oleate-induced HepG2 cells. **A** TG levels. **B** TC levels. **C** LDL-C levels. **D** Lipid accumulation after Oil Red O staining (scale bar = 500 nm)

HepG2 cells were incubated with 0.01 mM sodium oleate to obtain the NAFLD cell model. Lipid accumulation was evident in the model group, suggesting the successful establishment of the NAFLD model (Figs. 5, 6, *p* < 0.05). To examine the lipid-lowering effect of COS_2_ and butyric acid, the NAFLD cell model was subjected to different COS_2_ and NaB doses and incubated for 24 h. And the cell availability was detected (the data are shown in Additional file 1: Fig. S2). The Oil Red O staining results indicated that COS_2_ and NaB intervention significantly alleviated lipid accumulation (Figs. 5D and 6D), while only high COS_2_ doses (Fig. 5A, B, C, 0.2 mM, 0.5 mM, and 1 mM) reversed the TG, TC, and LDL-C levels in the NAFLD cell model. However, a less distinct lipid-lowering effect was evident in the model group at the lowest COS_2_ concentration (Fig. 5A, B, C, 0.02 mM). However, after NaB treatment, the TG, TC, and LDL-C levels decreased significantly, induced by sodium oleate (Fig. 6, *p* < 0.05).

### ***COS***_***2***_*** and NaB restored lipolysis in sodium oleate-induced HepG2 cells***

**Fig. 7:**
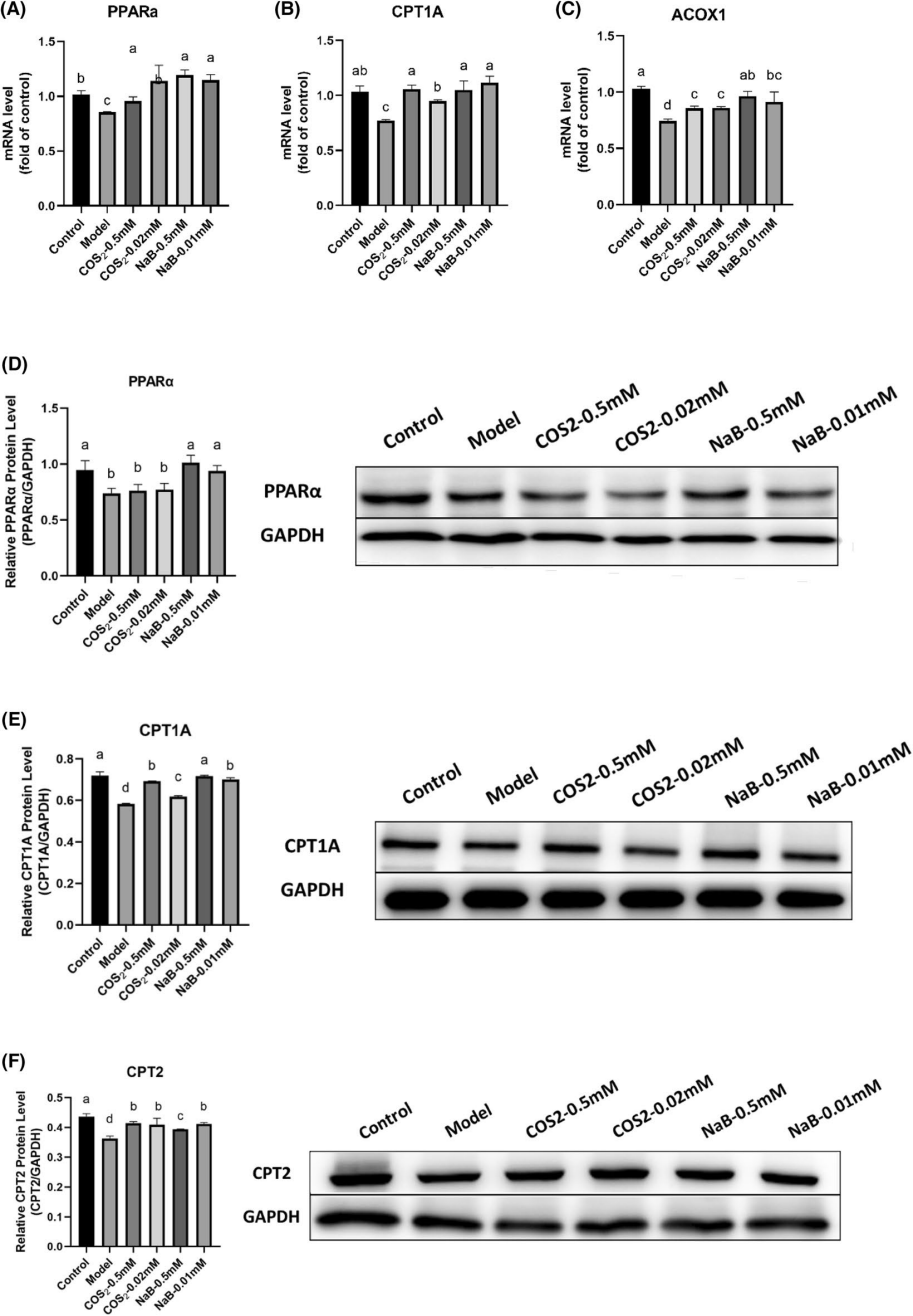
The mRNA and protein expression levels of the lipid β-oxidative metabolism pathway in sodium oleate-induced HepG2 cells, regulated by COS_2_ and NaB

The mRNA levels of the lipid β-oxidation pathway involving PPARα and its downstream targets, CPT1A and ACOX1, were inhibited in the NAFLD model compared to the control group (Fig. 7A, B, C, p < 0.05). All NaB doses and high COS_2_ doses elevated the mRNA levels of PPARα, indicating potential lipolysis stimulation, while low COS_2_ doses showed less effects to activate PPARα gene levels compared to the model group (Fig. 7A, p < 0.05). Therefore, low COS_2_ doses were less successful than NaB in reversing the PPARα protein levels (Fig. 7D). Similarly, all NaB doses and high COS_2_ doses were more effective in reversing CPT1A gene and protein levels, while low COS_2_ concentrations were less successful in increasing CPT1A (Fig. 7B, E). Furthermore, all COS_2_ and NaB doses restored CPT2 protein expression (Fig. 7F). Moreover, ACOX1, a rate-limiting enzyme involved in FFAs β-oxidation, had been more facilitated by NaB than COS_2_ in mRNA levels (Fig. 7C). Consequently, NaB was superior to COS_2_ at low concentrations in promoting oxidative lipid metabolism. Therefore, the butyric acid in the liver–gut axis may exert a more positive effect, warranting further attention.

### ***Structures of COS***_***2***_*** and butyric acid bound to PPARα***

**Fig. 8:**
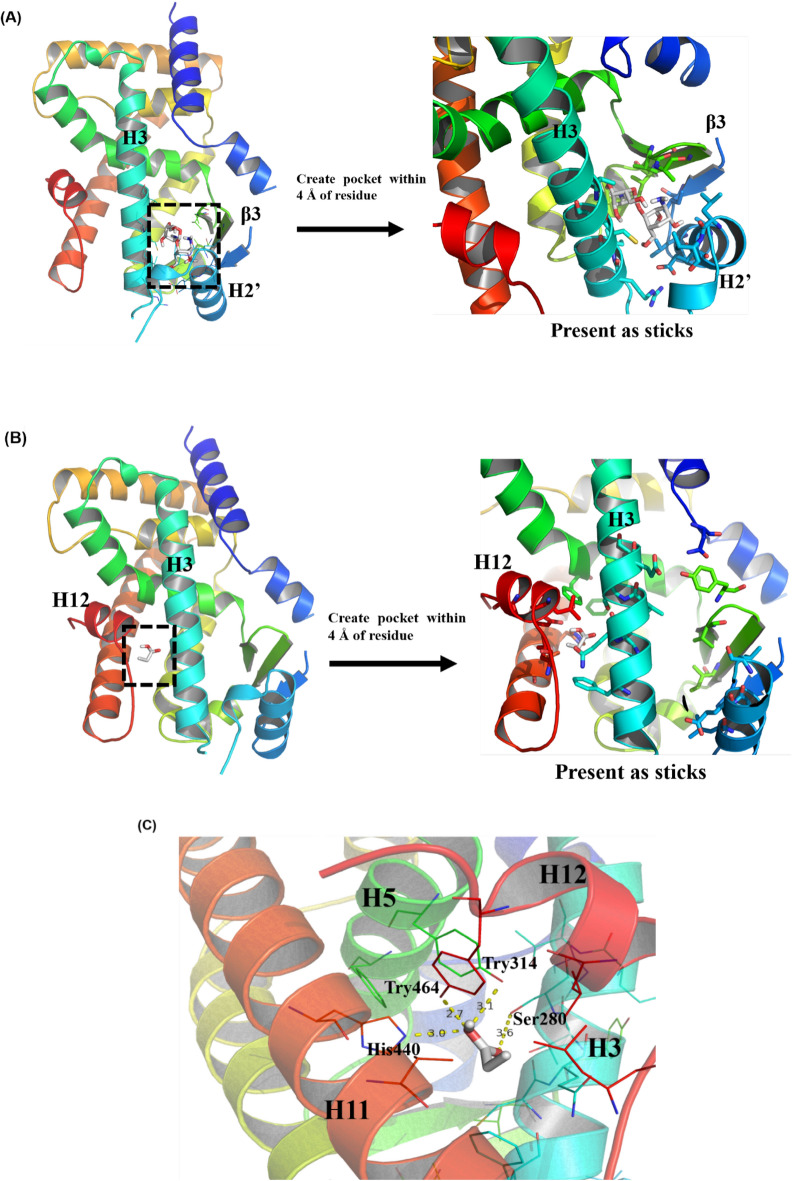
Docking COS_2_ and NaB in LBDs of PPARα. **A** COS_2_ and **B** butyric acid binding to pocket within 4 Å of residue of PPARα. **C** Hydrogen bond interaction of butyric acid and PPARα

PPARα involves in fatty acid metabolism and can be activated by ligands binding to its LBDs (Oyama et al. 2009). The LBDs of PPARα including hydrophilic, hydrophobic and amphiphilic pockets forming Y-shape domains (Han et al. 2020). The optimal docking modes of COS_2_ suggested that the ligand mainly contact with H3, H2’ helix and β3 strand at the hydrophobic pocket of PPARα, which is the entrance of the LBDs (Fig. 8A). While due to the size and hydrophily of butyric acid, the hydrophilic pocket between Helix 3 and Helix 12 including the activation factor-2 (AF-2) domain was occupied and hydrogen bonds were formed with amino acid residues Tyr464 on Helix 12, Try314 on Helix 5, Ser280 on Helix 3 and His440 on helix 11 (Fig. 8B, C). These interactions stabilized AF2 helix to recruit co-activators of receptor and facilitated the transcriptional activity of PPARα (Capelli et al. 2016; Han et al. 2020; Xu et al. 2001). Thus, these results well explained that butyric acid could be more effective than COS_2_ at low concentrations in promoting PPARα expression and stimulating oxidative lipid metabolism.

## Discussion

This study proposed that the lipid-lowering mechanism of COS_2_ was related to a specific metabolite, butyric acid. COS_2_ effectively alleviated lipid dysfunction by regulating the mitochondrial β-oxidation pathways in ob/ob^−/−^ mice. Additionally, COS_2_ exhibited a prebiotic effect by facilitating the accumulation of butyric acid, a specific metabolite with the potential to relieve hepatic lipid abnormality more effectively. Therefore, this study was extended to examine the lipid-lowering mechanism of COS_2_.

NAFLD diagnosis typically occurs when lipid accumulation accounts for 5% of the weight of the liver. Lipid metabolism dysbiosis represents the main pathogenic factor of NAFLD and can manifest in various ways, such as lipid uptake, de novo synthesis, and oxidative metabolism. It is been proved that COS_2_ can be absorbed and circulated through blood and exhibited its effects in liver (Chen et al. 2022). Our previous studies showed that COS_2_ downregulated hepatic lipogenesis-related targets and reduced lipid uptake (Shen et al. 2021). In this study, we found that COS_2_ enhanced the FFA oxidation process in the liver to reverse NAFLD lipid accumulation in the ob/ob^−/−^ mice. Mitochondrial FFA β-oxidation represents the main lipid oxidation pathway. The upstream regulator of lipid metabolism is FXR, a ligand-activated receptor belonging to the nuclear receptor superfamily and essential for regulating FFA β-oxidation by controlling PPARα (Proctor et al. 2006; Kast et al. 2001; Li et al. 2021; Xi et al. 2020). COS_2_ improved FXR gene expression, activating liver lipolysis (Fig. 3A). PPARα, the downstream target of FXR, represents the key enzyme that controls the FFA β-oxidative system. As shown in Fig. 3C, high COS_2_ doses significantly upregulated the PPARα mRNA levels. Moreover, the mitochondrial entry process represented the FFA β-oxidation rate-limiting step and mainly occurred via ACSL1, CPT1A, and CPT2 catalysis. The FFAs were activated to form acyl-CoA via ACSL1 (Huh et al. 2020), which was transferred successfully into the mitochondria with the help of CPT1A and CPT2, finally initiating the β-oxidation process (Xi et al. 2020). COS_2_ promoted the expression of gene and protein levels of ACSL1, CPT1A, and CPT2 to facilitate the rate-limited process of lipolysis, demonstrating the internal mechanism of COS_2_ in improving hepatic lipid metabolism of NAFLD in ob/ob^−/−^ mice.

In addition, COS_2_ has been found to significantly increase the production of butyric acid by enriching the microbial communities in the intestinal microenvironment. In an in vitro anaerobic fermentation study using fecal samples from healthy individuals, COS_2_ was shown to promote the growth of functional microbial communities, such as *Clostridium_butyricum*, *Clostridium*, and *Parabacteroides*, which are known to produce substantial amounts of butyric acid (Ji et al. 2022). Similarly, in the fermentation of fecal samples obtained from patients with NAFLD, COS_2_ enriched the core functional microbial communities responsible for butyric acid production, including *Clostridium *sensu stricto* 13*, *Parabacteroides*, *Romboutsia*, *Holdemanella*, *Bacteroides*, *Bacterium NLAE zl_G201*, *Erysipelatoclostridium*, and *Lactococcus* (Ji et al. 2021). Moreover, COS_2_ and COS_3_ were found to promote the growth of intestinal probiotics such as *Bifidobacterium* and *Lactobacillus* in *ob/ob* model mice and significantly increase the abundance of functional microbial communities, such as *Akkermansia*, *Clostridiales*, *Faecalibaculum*, *Roseburia*, *Ruminiclostridium*, and *Alistipes*, which are also known to produce butyric acid. Previous study reported that butyrate had been reported to induce the reduction of lipid accumulation (Zong et al. 2023). For the intervention mechanism investigation, NaB-induced PPARα activation stimulates fatty acid β oxidation, thus contributing to amelioration of high-fat diet-induced NAFLD in adult rats (Sun et al. 2018). Thus, enterohepatic circulation is possibly responsible for the COS_2_ anti-NAFLD effect.

Due to the complexity of NAFLD pathogenesis, the following hypotheses could explain the anti-NAFLD impact of COS_2_: 1. COS_2_ alleviated hepatic lipid accumulation via direct liver lipid homeostasis. 2. COS_2_ increased the butyrate levels, producing a distinct hepatic anti-hyperlipidemic effect via enterohepatic circulation. To confirm this, HepG2 cell experiments were established using sodium oleate to induce lipid accumulation and create a NAFLD cell model. The metabolic kinetics study results of COS_2_ revealed a physiological concentration of 0.02 mM in the serum of the rats after intragastric administration of 500 mg kg^−1^. The physiological butyric acid concentration was around 3 μM in human serum (Behary et al. 2021) and between 26 μM to 48 μM in the portal serum of mice (Jakobsdottir et al. 2013). Therefore, 0.02 mM COS_2_ and 0.01 mM NaB were selected as the low dose interventions in the NAFLD cell model, showing that both treatments displayed anti-hyperlipidemia activity. However, at the physiological concentration, NaB was more successful in restoring the lipid levels, as well as the gene and protein expression of PPARα, CPT1A, ACOX1, and CPT2 than COS_2_. Moreover, in molecular docking analysis, the results showed that COS_2_ interacted with PPARα in hydrophobic pocket and stuck in the entrance of LBDs of PPARα protein. While butyric acid could crush into the hydrophilic pocket and form hydrogen bonds with AF2 domain, which was important for the receptor to activate the transcriptional activity (Xu et al. 2001). Therefore, butyric acid was superior in stimulating FFA β-oxidation and preventing lipid accumulation. The intestinal microenvironment and enterohepatic circulation status are crucial for NAFLD remission. COS_2_ accelerated the FFA β-oxidation pathway by promoting butyric acid production in the intestinal tract, reaching the liver via enterohepatic circulation, and binding to the FFA β-oxidation targets PPARα to improve lipid metabolism abnormalities.

## Conclusion

COS_2_ was found to induce the FFA β-oxidation pathway, which mitigated NAFLD by regulating gut microenvironment, particularly butyric acid metabolism, and its interaction with hepatic lipid metabolism. The results provide new insights into the mechanism of COS_2_ in lipid-lowering effects.

### Supplementary Information

**Additional file 1: Figure S1.** The HPLC-ELSD chromatogram of COS_2_. **Figure. S2.** The cell viability of COS_2_ and NaB treatment in SO-induced NAFLD cell model. **Table S1.** Primers of the target sequence.

## Data Availability

All data generated or analyzed during this study are included in this published article.

## Abbreviations

COS_2_: Chitobiose
NAFLD: Non-alcoholic fatty liver disease
LBDs: Ligand binding domains
NASH: Non-alcoholic steatohepatitis
COS: Chitosan oligosaccharides
TG: Triglyceride
FFAs: Free fatty acid
CPT1A: Carnitine palmitoyl transferase 1A
CPT2: Carnitine palmitoyl transferase 2
SCFAs: Short-chain fatty acids; NaB: Sodium butyrate
TC: Total cholesterol
LDL-c: Low-density lipoprotein cholesterol
HDL-c: High-density lipoprotein cholesterol
SPF: Specific-pathogen-free
PVDF: Polyvinylidene difluoride
TBST: Tris-buffered saline with tween 20
FXR: Farnesoid X receptor
ACSL1: Acyl-CoA synthetase long-chain family member 1
