# Supplementary material for: Chitobiose exhibited a lipid-lowering effect in ob/ob−/− mice via butyric acid enrolled liver–gut crosstalk
Source: Bioresour Bioprocess. 2023 Nov 9;10(1):79. doi: 10.1186/s40643-023-00696-7 (PMC10991647; doi:10.1186/s40643-023-00696-7)
Supplement: Supplementary file 1 — Additional file 1: Figure S1. The HPLC-ELSD chromatogram of COS2. Figure. S2. The cell viability of COS2 and NaB treatment in SO-induced NAFLD cell model. Table S1. Primers of the target sequence. [file 40643_2023_696_MOESM1_ESM.docx]

**Additional file**

**
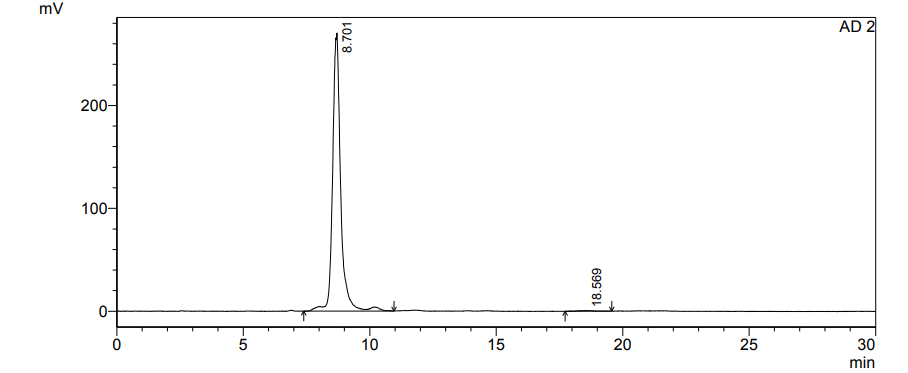
**

**Fig. S1 The HPLC-ELSD chromatogram of COS_2_**


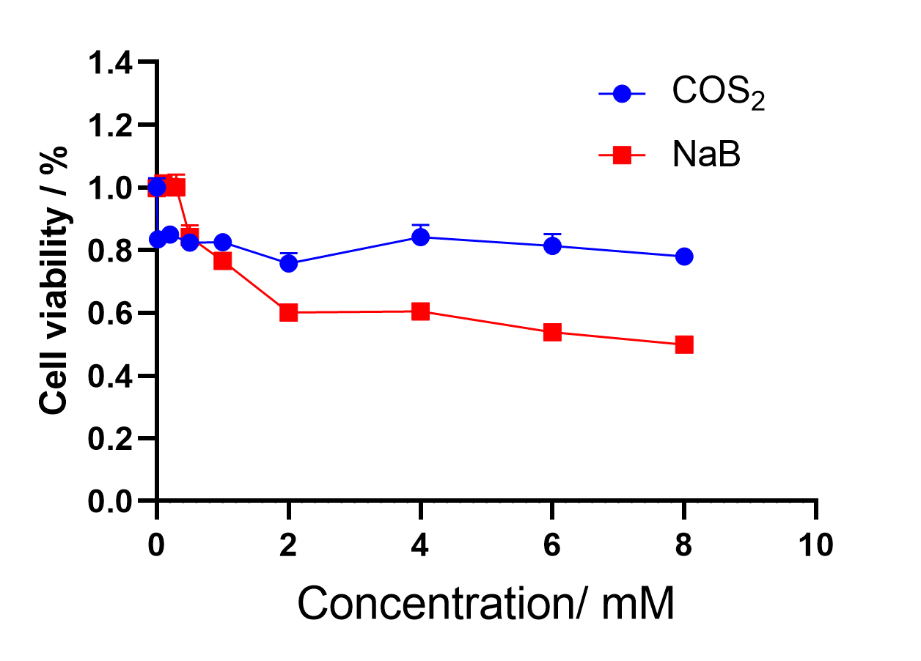


**Fig. S2 The cell viability of COS_2_ and NaB treatment in SO-induced NAFLD cell model**

**Supplementary Table S1** Primers of the target sequence

| Gene | Species | Forward (5' to 3') | Reverse (5' to 3') |
| --- | --- | --- | --- |
| β-actin | mice | AACACCCCAGCCATGTACG | ATGTCACGCACGATTTCCC |
| FXR | mice | GGCAGAATCTGGATTTGGAATCG | GCCCAGGTTGGAATAGTAAGACG |
| ACSL1 | mice | CACTTCTTGCCTCGTTCCAC | GTCGTCCCGCTCTATGACAC |
| PPARα | mice | TCATCAAGAAGACCGAGTCC | CCTCTTCATCCCCAAGCGTA |
| CPT1A | mice | ATGGCAGAGGCTCACCAAGC | GATGAACTTCCAGGAGTGC |
| CPT2 | mice | GCTCCGAGGCATTTGTCA | CCCATCGCTGCTTCTTTG |
| ACOX1 | mice | GCCTGCTGTGTGGGTATGTCATT | GTCATGGGCGGGTGCAT |
| GAPDH | human | CCATGGAGAAGGCTGGG | CAAAGTTGTCATGGATGACC |
| PPARα | human | GGTGGACACGGAAAGCCCAC | GGACCACAGGATAAGTCACC |
| CPT1A | human | GATTTCCATTCCTTCCCATTCG | CTCGTATGTGAGGCAAAACTTG |
| ACOX1 | human | CGGAAGATACATAAAGGAGACC | AAGTAGGACACCATACCACCC |
